# Supplementary material for: Acoustomicrofluidic assembly of oriented and simultaneously activated metal–organic frameworks
Source: Nat Commun. 2019 May 23;10:2282. doi: 10.1038/s41467-019-10173-5 (PMC6533252; doi:10.1038/s41467-019-10173-5)
Supplement: Supplementary file 1 — Supplementary Information [file 41467_2019_10173_MOESM1_ESM.pdf]

Description of the Supplementary File

File name: Ahmed_SupplementaryInformation.docx

Title: Acoustomicrofluidic Assembly of Oriented and Simultaneously Activated Metal-Organic Frameworks

Authors: Ahmed et al.

Description: Supplementary Figures and Table

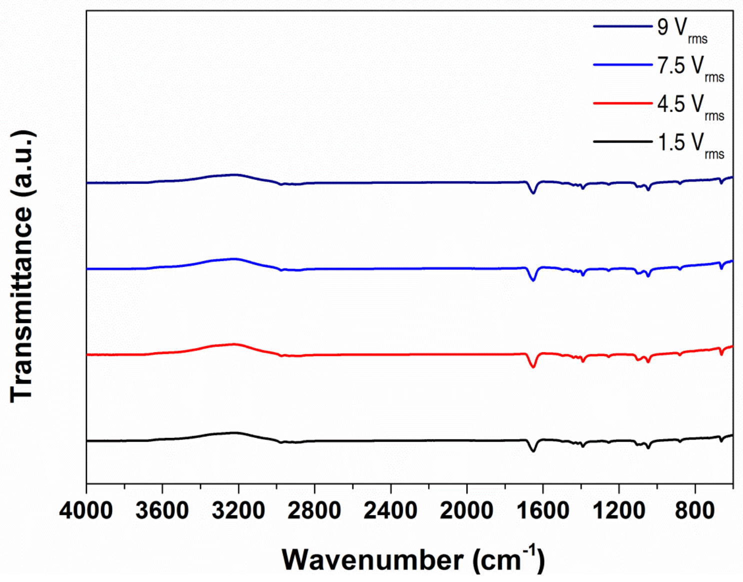

Supplementary Figure 1 Fourier Transform Infrared (FTIR) analysis. FTIR spectra of HKUST-1 crystals synthesized at different input voltages.

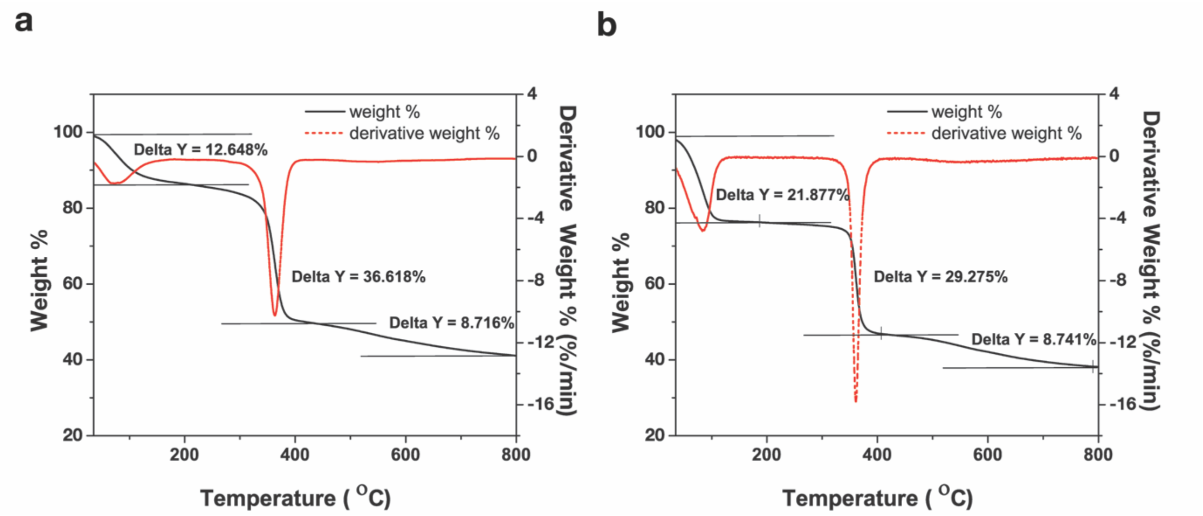

Supplementary Figure 2 Thermal gravimetric analysis (TGA). TGA curves for (**a**) HKUST-1 synthesized at 9 V_{rms} compared to that for (**b**) bulk HKUST-1 synthesized by spontaneous evaporation.

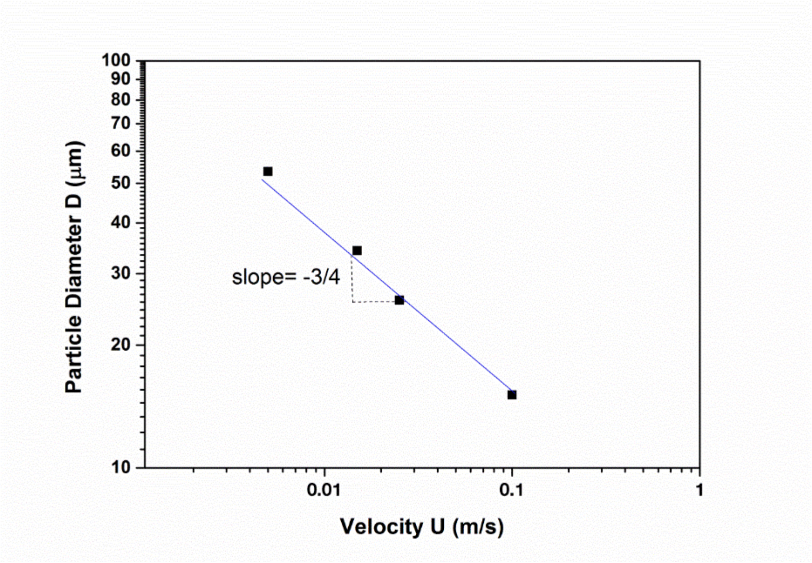

Supplementary Figure 3 Crystal Dimension. Crystal diameter *D* as a function of the increasing acoustic energy and hence convective velocity in the drop *U*, which is in good agreement with the theoretical -3/4 slope associated with the Kolmogoroff length scale for the characteristic eddy dimension (solid blue line), suggesting the likelihood that the crystal growth is limited by their confinement within the localized vortices arising due to the acoustically-driven turbulent convective flow within the drop.

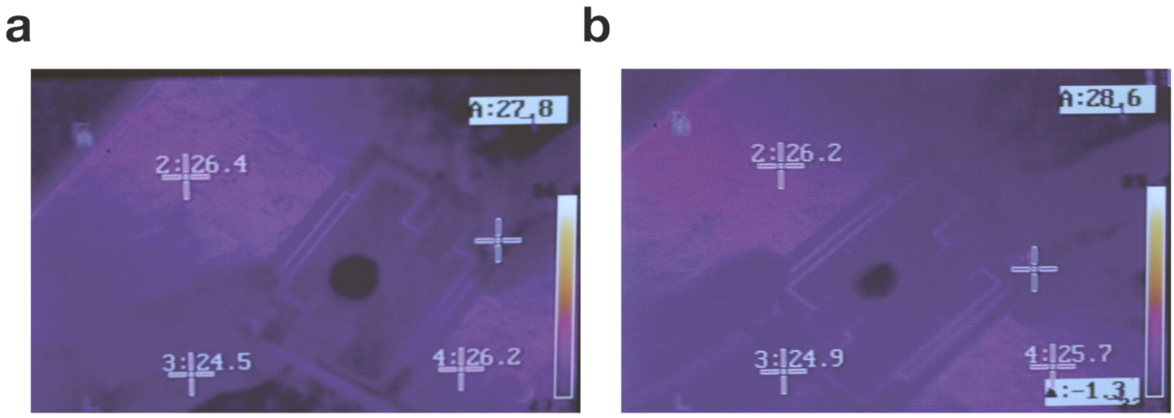

Supplementary Figure 4 Thermal measurements. Temperature measurements of a sessile drop on the SAW device, (**a**) in the absence, and, (**b**) in the presence (9 V_{rms}) of the acoustic excitation. The miniscule temperature rise (temperatures shown in °C) due to the SAW irradiation suggests that heating effects on the evaporation of the crystallizing drop due to the acoustic forcing are negligible.

Supplementary Table 1 Crystal dimension. Lateral size distribution of HKUST-1 crystals at different input voltages for the acoustic signal compared to the control (No SAW) in which there is no acoustic excitation.

No SAW		1.5 V_{rms}		4.5 V_{rms}		7.5 V_{rms}		9 V_{rms}	
Lateral Dimension (µm)	Frequency	Lateral Dimension (µm)	Frequency	Lateral Dimension (µm)	Frequency	Lateral Dimension (µm)	Frequency	Lateral Dimension (µm)	Frequency
31.92347538	5	31.92347538	8	15.96173769	3	15.96173769	3	4.223078842	3
46.53606	5	46.53606231	15	22.57330592	7	22.57330592	17	5.972335373	1
56.4332648	7	56.4332648	10	33.85995888	14	29.86167687	3	7.226984113	2
64.836885	8	64.836885	8	37.43359301	4	33.85995888	4	8.37040586	1
73.14587118	12	73.14587118	6	40.69460597	3	37.43359301	4	9.375398081	1
85.95658807	8			45.14661184	5	40.69460597	3	10.28263021	1
92.38523609	1			47.88521307	2	43.71301895	3	11.11606401	1
108.2577721	1			50.47544651	3	47.88521307	4	12.56828482	1
				52.93909492	2	50.47544651	3	13.25881512	1
				55.29308131	3	52.93909492	2	14.5418351	1
				58.64716912	3	58.64716912	2	15.14263395	17
				64.836885	6	60.78048631	2	16.23866582	1
				67.71991776	3	66.77273939	2	17.81003796	1
				75.71316977	3	73.14587118	2	18.26904381	1
								19.2204774	1
								20.1269854	1
								21.38515468	1
								22.57330592	1
								23.72882898	1
								24.44283595	2
								25.16189617	4
								27.18185982	2
								33.55763177	2
								34.58581131	1
								36.55551576	2
								37.5185729	1
								40.88199579	3
								42.15530884	2
								44.77829319	2
								45.74924732	1
								46.5086801	1
								52.552673	6
								53.04726891	1
								54.67914518	1
								55.32762869	1
								57.05065355	2
								58.57109567	1
								59.75533982	1
								61.64414003	2
								63.60707408	2
								64.29430738	1
								64.836885	2
								72.52497922	4
